# Lectures on the Treatment of the Diseases of the Kidneys and the Bladder

**Published:** 1881-09

**Authors:** E. L. Schurly

**Affiliations:** Professor of the Practice of Medicine in Detroit Medical College, late Professor of Materia Medica and Therapeutics in the same college


ARTICLE II.

### LECTURES ON THE TREATMENT OF THE DISEASES OF THE KIDNEYS AND THE BLADDER.

BY PROF. E. L. SCHURLY, M. D.,

Professor of the Practice of Medicine in Detroit Medical College, late Professor of Materia Medica and Therapeutics in the same college. Reported by HUGO ERICHSEN.

GENTLEMEN—If the arterioles of the kidneys are contracted, it would be folly to select broom or digitalis for a diuretic. Buchu has a direct action on the secreting apparatus of the kidneys. Digitalis and broom are used where we have a low blood pressure and transudation of fluid from the blood. Uva ursi and sweet spirits of nitre are kidney stimulants. If we desire to wash out the renal system we select a saline diuretic as citrate of potassium. This may be combined with nitrate of potassium. We only use digitalis when the blood pressure is low, as in the scarlatina or anasarca. Sometimes we combine citrate or nitrate of potassium with digitalis. Even if we have mechanical obstructions in the kidneys, diuretics are given to render the urine non-irritating. Skimmed milk (least irritating) is the best diuretic for these cases.

*Nephritic Colic* is caused by malarial and lead poisoning, and by the overuse of alcohol.

*Symptoms.*—Pain along the course of the ureter.

*Treatment.*—Give a cholagogue cathartic, as ten grains each of jalap and calomel, if constipation is present, and follow this with rochelle salts. Rhubarb should not be given in these cases, because it contains oxalates. If much pain be present, the first thing we have to treat is the pain. Give morphia per orem or hypodermically, with or without atropia. Chloroform may be used. In the summer a hot bath often gives relief. The attending physician ought to stay at the bedside of his patient till one or more of the urate crystals have passed. Do not give too much morphine, because after the patient has passed the crystals, reaction sets in and he may die from opium narcosis. Chloroform and ether are preferable to morphia. The next thing to do is to prevent future attacks. Lay down rules of life. Forbid leguminous vegetables, cabbage, etc., alcohol and all kinds of pastry. Do not direct the patient's attention to his or her urine. Triticum repens and buchu are useful. If the attacks occur frequently and are due to derangement of the hepatic functions the patient becomes hypochondriacal.

*Oxaluria.*—An excessive deposit of oxalate of lime. In females this disease is often due to tipping over of the uterus.

*Treatment.*—Hydrobromic acid (dilute) and the mineral acids will prove useful. Permanganate of potassium is given. Sudden fright may bring on a cure. Influence the patient's mind.

*Congestion of the Kidneys.*—This disease is generally brought on by taking cold.

*Treatment.*—The first indication is rest. Put the patient to bed. A hot poultice may be applied to the region of the kidney. A differential diagnosis must be made in these cases between congestion of the kidney and myalgia of the quadratus lumborum. We often have hæmaturia, especially if the disease is due to shock and injury. In this case we use astringents. We give a saline cathartic followed by a diaphoretic. Acetate of ammonia is useful.

*Acute Inflammation of the Kidney.*—Cut off all diet. Let the patient take water only for one day. If water dyspepsia comes on it can easily be removed by a steam bath. If you desire to blister in the lumbar region, apply a mustard plaster. Rest is very important. Every other day you may give a cathartic.

*Catarrh of the Bladder.*—To rest the bladder we give urethral supositories of carbolic acid and gum acacia or rectal or vaginal supositories containing opium. Hot sitz baths or hot poultices are useful. In the female we may add to this a vaginal injection. We must prevent fermentation in the bladder. We must render the urine alkaline by giving lemonade, citric acid, etc.

*Chronic Vesical Catarrh.*—Enlargement of the prostate gland may take place at the age of forty. Urine is retained and a catarrh set up on account of this enlargement. It also can be caused by gleet and stricture. It is often caused by alcoholic drinks and gout.

*Treatment.*—Local and general. We use mild diuretics (buchu, triticum, repens, etc.) to wash out the bladder. We may give distilled or rain water for diuretic purposes. The patient must avoid all vegetables containing oxalates. Limit the supply of meat and fat.

*Local Treatment.*—A flexible catheter should be passed into the bladder once a day. If much blood and mucus comes away, wash out the bladder with a fountain syringe. You may add benzoic acid to the water. The benzoic acid decomposes in the bladder and forms hippuric acid, which is a sedative.

℞ Acidi benzoici, . . . . . . gr iij.
Aquæ, . . . . . . . . ℥ j.

Borax injections are useful. Give morphia, if necessary.

